# Exploring the Gut–Mitochondrial Axis: p66Shc Adapter Protein and Its Implications for Metabolic Disorders

**DOI:** 10.3390/ijms25073656

**Published:** 2024-03-25

**Authors:** Ana Clara da C. Pinaffi-Langley, Elizabeth Melia, Franklin A. Hays

**Affiliations:** 1 Department of Nutritional Sciences, College of Allied Health, University of Oklahoma Health Sciences, Oklahoma City, OK 73117, USA; anaclara-dacostapinaffilangley@ouhsc.edu (A.C.d.C.P.-L.); elizabeth-melia@ouhsc.edu (E.M.); 2 Stephenson Cancer Center, University of Oklahoma Health Sciences, Oklahoma City, OK 73117, USA

**Keywords:** p66Shc, adaptor protein, oxidative stress, gut microbiota, inflammation, mitochondrial dysfunction

## Abstract

This review investigates the multifaceted role of the p66Shc adaptor protein and the gut microbiota in regulating mitochondrial function and oxidative stress, and their collective impact on the pathogenesis of chronic diseases. The study delves into the molecular mechanisms by which p66Shc influences cellular stress responses through Rac1 activation, Forkhead-type transcription factors inactivation, and mitochondria-mediated apoptosis, alongside modulatory effects of gut microbiota-derived metabolites and endotoxins. Employing an integrative approach, the review synthesizes findings from a broad array of studies, including molecular biology techniques and analyses of microbial metabolites’ impacts on host cellular pathways. The results underscore a complex interplay between microbial metabolites, p66Shc activation, and mitochondrial dysfunction, highlighting the significance of the gut microbiome in influencing disease outcomes through oxidative stress pathways. Conclusively, the review posits that targeting the gut microbiota-p66Shc–mitochondrial axis could offer novel therapeutic strategies for mitigating the development and progression of metabolic diseases. This underscores the potential of dietary interventions and microbiota modulation in managing oxidative stress and inflammation, pivotal factors in chronic disease etiology.

## 1. Introduction

Mitochondria are membrane-bound organelles responsible for generating most of the chemical energy (in the form of adenosine triphosphate [ATP]) needed to support normal cellular function. In addition to their familiar “powerhouse” trait, mitochondria are also critically involved in redox signaling and calcium homeostasis. Every human cell–except mature erythrocytes–contains mitochondria, with the proportion varying according to cell-specific metabolic demands. It is unsurprising then, that mitochondrial dysfunction is implicated in the etiology of several diseases, including neurodegenerative diseases and highly prevalent noncommunicable diseases such as type 2 diabetes and cardiovascular diseases [1,2].

The adaptor protein p66Shc is a redox sensor and oxidoreductase that plays a role in apoptosis and reactive oxygen species (ROS) production [3]. In particular, its localization in the mitochondria, which is increased during oxidative stress conditions, is a major driver of mitochondrial dysfunction. Since the recognition of p66Shc as a “lifespan regulator” [4], many studies have delved into elucidating its mechanistic pathways and cellular interactions. However, to date, there has been only one attempt to integrate the influence of the gut microbiota on p66Shc activation [5]. The understanding of how the gut microbiota and its metabolites are integrated into human health has inaugurated a more integrative approach to physiology [6,7,8]. Importantly, microbial metabolites are involved in redox signaling and the oxidative stress response [9,10], which, in turn, influences p66Shc activation. Therefore, this review aims to describe and explore oxidative stress as the link between gut microbiota, p66Shc, and mitochondrial dysfunction and related diseases. Understanding this link can open new strategies to utilize non-pharmacological interventions (for instance, diet modification) to modulate p66Shc activity and decrease mitochondrial dysfunction.

## 2. Overview of the ShcA Protein Family

**Figure 1:**
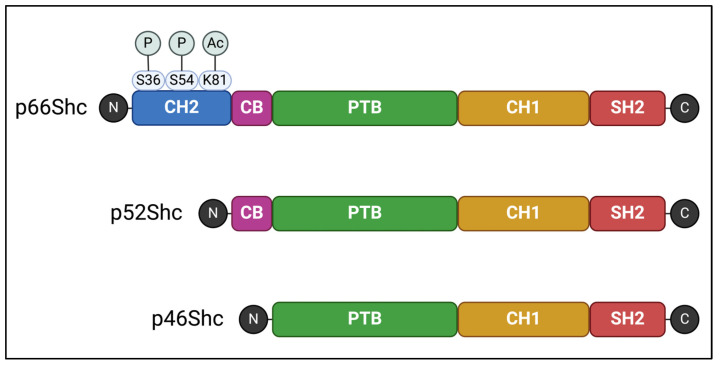
Functional domains of the ShcA proteins: collagen homology (CH1 and CH2), cytochrome C-binding (CB), phosphotyrosine-binding (PTB), and Src-homology (SH2) domains. Post-translation modifications highlighted in the CH2 domain of p66Shc are involved in its activity affecting reactive oxygen species production and apoptosis. Ser36 phosphorylation increases p66Shc translocation into mitochondria; Ser54 phosphorylation decreases p66Sch degradation by the proteasome; Lys81 acetylation increases Ser36 phosphorylation. P: phosphorylation, Ac: acetylation. Created with BioRender.com.

The Shc family comprises adaptor proteins that are critically involved in cellular signaling. In mammals, the ShcA protein sub-family is encoded by a single gene locus, *ShcA*. Using alternative splicing and different start codons, this locus encodes for three protein isoforms–p46Shc, p52Shc, and p66Shc. These proteins contain the same three functional domains: a phosphotyrosine-binding (PTB), a central collagen homology (CH1), and a C-terminal Src-homology domain. The shortest isoform, p46Shc, has the PTB domain as the N-terminal, whereas longer isoforms contain additional cytochrome C-binding domains. In addition, the longest isoform, p66Shc, has a second N-terminal collagen homology (CH2) domain [11,12] (Figure 1).

The functional domains of ShcA proteins undergo different post-translational modifications that modulate their activity. In p52Shc and p46Shc, tyrosine residues in the CH1 domain are phosphorylated in response to receptor tyrosine kinase activation. This event starts a signaling cascade resulting in the promotion of cellular survival, migration, and proliferation. Briefly, phosphorylated p52Shc or p46Shc recruited to an activated receptor tyrosine kinase binds to another adaptor protein, growth factor receptor-bound protein 2 (Grb2), which is constitutively associated with Son of Sevenless homolog 1 (Sos1), a guanine nucleotide exchange factor for Ras. Recruitment of the Grb2-Sos1 complex to the plasma membrane induces Ras activation, which subsequently stimulates signaling via the mitogen-activated protein kinase (MAPK) pathway [12,13].

Although p66Shc also binds to activated receptor tyrosine kinases, this interaction does not induce the MAPK signaling cascade. Instead, p66Shc activation induces ROS production and apoptotic pathways (discussed in further detail in the following section) [13]. p66Shc is also antagonistic to p46/p52Shc proteins. ShcA proteins are encoded by a common gene locus; thus, an increase in p66Shc expression begets a decrease in p46/p52Shc expression. Additionally, post-translational modifications in the extra CH2 domain of p66Shc are associated with increased cellular ROS levels [14,15,16], setting it firmly apart from the other ShcA proteins (Figure 1). Particularly, phosphorylation of Ser36 in response to oxidative stress signals increases p66Shc translocation into mitochondria, an early event leading to excessive mitochondrial ROS production and cell death [14]. Thus, p66Shc plays a crucial role in mitochondrial dysfunction and related pathologies (Table 1), with several reviews exploring the connections between p66Shc and specific diseases [17,18,19,20].

## 3. p66Shc and Oxidative Stress

**Figure 2:**
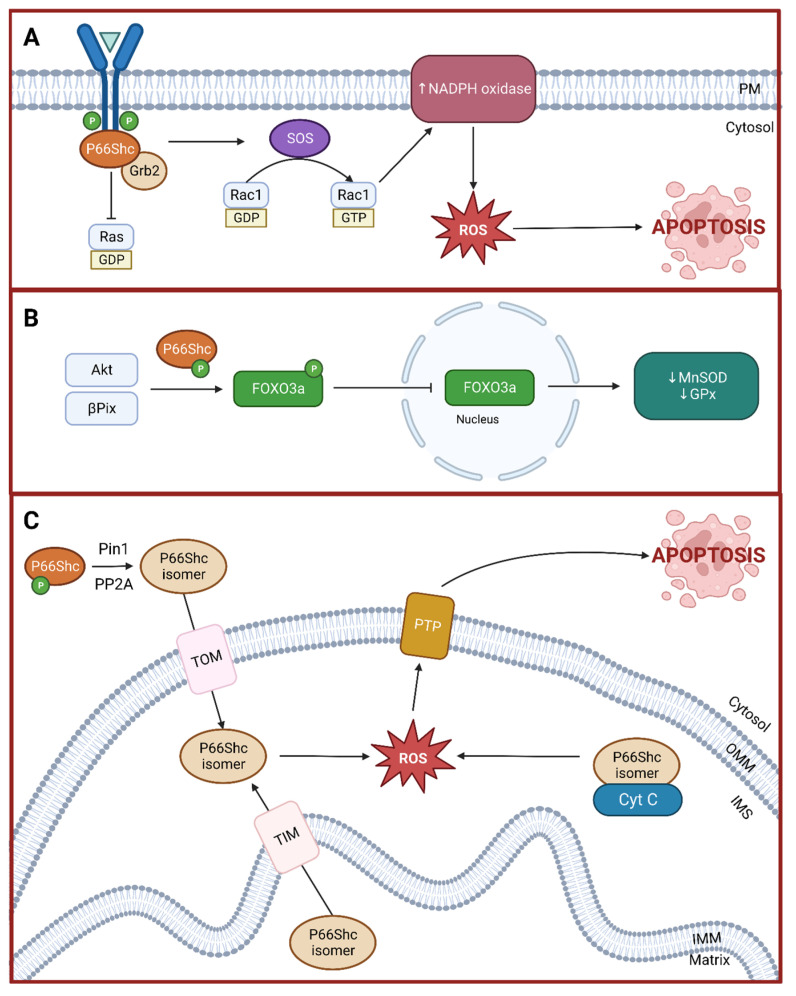
Schematic representation of p66Shc-mediated signaling pathways and their role in apoptosis. (**A**) Competitive inhibition of p52/p46Shc by p66Shc at the Grb2-SOS complex, resulting in the activation of Rac1 instead of Ras, leading to increased NADPH oxidase activity and subsequent reactive oxygen species (ROS) production, culminating in apoptosis. (**B**) Role of p66Shc in the inactivation of Forkhead-type transcription factors (FOXO3a) via Akt phosphorylation and βPix sequestration from the nucleus, which diminishes the expression of antioxidant enzymes such as MnSOD and GPx, further promoting apoptosis. (**C**) Role of p66Shc in mitochondrial apoptosis through its translocation into the intermembrane space mediated by Pin1 and PP2A, leading to increased mitochondrial ROS production and cytochrome c release, triggering the apoptotic cascade. Created with BioRender.com.

As mentioned above, p66Shc is associated with ROS production and apoptosis. This protein achieves these effects via three distinct mechanisms that are illustrated in Figure 2 and discussed below. However, before delving into these mechanisms, it is important to highlight that p66Shc is a redox sensor and its associated cellular outcomes are dependent on the local environment. The mechanisms discussed below are relevant when sustained and heightened (i.e., pathological) stress levels are present. Under normal physiological conditions, p66Shc responds to transient stress signals in a pro-survival and -proliferation manner [18,39,40].

### 3.1. Rac1 Activation

Under oxidative stress conditions, p66Shc is phosphorylated at the Ser36 residue in the CH2 domain by kinases such as protein kinase C-β and c-Jun N-terminal kinase [41]. This activated p66Shc competes with p52/p46Shc for binding to Grb2, causing its disassociation from Sos1. Sos1 is then free to associate with Eps8 and E3b1, forming a complex that activates Rac1 instead of Ras [42]. In this way, p52/p46Shc function is effectively inhibited and Ras-mediated activation of the MAPK pathway is disrupted. Further, active Rac1 increases p66Shc stability and activity while decreasing proteasomal degradation [43]. Finally, active Rac1 also promotes the activation of membrane-bound NADPH oxidase, thereby increasing intracellular ROS levels [44].

### 3.2. Forkhead-Type Transcription Factors Inactivation

Forkhead-type transcription factors, particularly those in the O subgroup (FoxO), are involved in the cellular stress response by modulating the expression of antioxidant enzymes such as catalase and superoxide dismutase [45,46]. FoxO proteins are negatively regulated by serine/threonine protein kinase B (Akt). When activated, Akt phosphorylates FoxO proteins, which causes them to be exported out of the nucleus, precluding their transcriptional activity [46]. Akt is activated by various stimuli such as insulin and growth factors. Under oxidative stress stimulus, activated p66Shc mediates Akt activation and subsequent FoxO sequestration from the nucleus [47,48]. p66Shc may also inactivate FoxO proteins in an Akt-independent manner via complexation with βPix [49,50]. In this way, p66Shc decreases the availability of endogenous antioxidants.

### 3.3. Mitochondria-Mediated Apoptosis

Under normal physiological conditions, p66Shc is distributed in the cytoplasm (32%), endoplasmic reticulum (24%), and mitochondria (44%) [51]. p66Shc in the cytoplasm remains inactive until stress signals induce p66Shc activation and increase trafficking into the mitochondria. Briefly, prolyl isomerase 1 (Pin1) interacts with active p66Shc, mediating a cis–trans isomerization. This change in conformation allows protein phosphatase 2A (PP2A) to interact with p66Shc and dephosphorylate the Ser36 residue. These consecutive changes facilitated by Pin1 and PP2A are necessary for p66Shc to interact with the outer membrane translocase and reach the intermembrane space [14]. In addition, stress signals also mediate the translocation of the small fraction of p66Shc normally present in the matrix into the intermembrane space via interaction with the intermembrane translocase [51,52]. This excessive p66Shc translocation into the mitochondrial intermembrane space increases local ROS levels and starts a cascade of signals leading to apoptosis.

One of the ways p66Shc increases mitochondrial ROS levels is through its oxidoreductase activity, thereby being a source of superoxide anion [39]. This activity is mediated by intramolecular interactions between cysteine residues in the CH2 and PTB domains as well as a key tyrosine residue (Tyr10) in the CH2 domain [39]. However, the full mechanism of how p66Shc generates superoxide anions remains unknown. p66Shc also interacts with cytochrome C peroxidase to increase mitochondrial ROS levels. Cytochrome C peroxidase is part of the antioxidant machinery responsible for converting ROS into harmless products such as water. Although cytochrome C peroxidase activity is increased in response to escalating ROS levels, p66Shc inhibits its activity, thereby further blunting antioxidant defenses (see FoxO inactivation above). Prolonged oxidative stress inside the mitochondria causes a wide array of deleterious outcomes including cytochrome C disassociation, mitochondrial DNA damage, electron transport chain disruption, impaired antioxidant defenses, and ultimately permeability transition pore formation. Disassociated cytochrome C escapes into the cytoplasm through this pore, thus starting a caspase signaling cascade that leads to cell death [18].

## 4. Gut Microbiota and Oxidative Stress

The gut microbiota is a complex ecosystem of trillions of microorganisms including bacteria, viruses, yeast, protozoa, and fungi. These microorganisms reside in the gastrointestinal tract, primarily within the colon, and play an indispensable role in human physiology. The gut microbiota contributes to various processes such as digestion, immune response training and homeostasis, bioactive compound biosynthesis, toxin elimination, cell proliferation, among others [53,54]. Gut microbes influence their local environment as well as distal tissues and cells via microbial patterns (e.g., toll-like receptor [TLR] ligands) and a variety of metabolites. These microbial metabolites can be derived from dietary components (e.g., short-chain fatty acids [SCFA], tryptophan catabolites, trimethylamine-*L*-oxide) or from host metabolism (e.g., secondary bile acids), or synthesized de novo by the microbes themselves (e.g., branched-chain amino acids, bacterial vitamins) [8,55]. Thus, the metabolic output of the gut microbiota represents its main mode of communication with the host.

A diverse population of microorganisms, primarily consisting of obligate anaerobes such as Firmicutes and Bacteroidetes, allows for a health-promoting and functional environment primed for carrying out efficient physiologic processes. Conversely, conditions that promote the proliferation of facultative anaerobes decrease microbial diversity and increase local and systemic inflammation. This alteration in the gut microbiota community ultimately changes their metabolic output toward disease-promoting signals [54,56,57]. In fact, several pathophysiological conditions have been associated with the gut microbiota and its influence on inflammation and oxidative stress (Table 2).

In normal physiology, commensal bacteria stimulate transient ROS production in the gut, which is essential for cell proliferation and motility as well as inflammation and immune response [68,69,70]. They achieve this by shedding microbial patterns such as small formylated peptides that are recognized by pattern recognition receptors like formyl peptide receptors. These receptors increase the activity of NADPH oxidases [71], which generate ROS and activate redox sensor proteins and associated signal transduction pathways. For instance, Ubc12–a Nedd8 ligase involved in NF-κB activation–is inactivated in these conditions [72,73]. This mechanism likely mediates host immune tolerance to the gut microbiota. However, excessive and sustained ROS production can lead to detrimental immune suppression and downregulation of survival pathways.

Commensal bacteria also limit oxidative conditions in the gut. Butyrate-producing bacteria, such as those belonging to the Firmicutes phylum, stimulate the peroxisome proliferation activated receptor-gamma (PPAR-γ) pathway and β-oxidation in intestinal cells [74]. PPAR-γ signaling shifts cellular energy production toward oxidative phosphorylation, thereby stimulating oxygen consumption, and preventing it from translocating into the intestinal lumen. Depletion of SCFA-producing bacteria and subsequent down-regulation of the PPAR-γ pathway favors anaerobic glycolysis for energy production. Consequently, underutilized oxygen reaches the intestinal lumen, conferring a survival and proliferation advantage to facultative anaerobes such as *Escherichia*, *Salmonella*, and other genera of the *Enterobacteriaceae* family [75].

The proliferation of pathogenic members of the *Enterobacteriaceae* family is a driver of systemic inflammation and oxidative stress. *Enterobacteriaceae* are Gram-negative bacteria and, as such, they shed endotoxins termed lipopolysaccharides (LPS). LPS are components of Gram-negative bacterial outer membranes comprised of a hydrophobic domain (lipid A), a polysaccharide core, and an oligomeric polysaccharide tail (O-antigen) [76]. In a healthy gut, the intestinal barrier (formed by an intact epithelial cell layer and a mucus layer) will prevent LPS present in the luminal side from translocating into the basal side, where it can interact with pattern recognition receptors and induce inflammatory responses. However, alterations in the bacterial community and high levels of LPS disrupt intestinal barrier integrity, thus resulting in what is often termed a “leaky gut” [77,78]. This allows LPS to escape the intestinal lumen and enter systemic circulation.

While LPS shed by commensal bacteria can be beneficial to host metabolism [79], pathogenic bacteria cast off LPS are associated with metabolic endotoxemia, a condition characterized by chronic low-grade inflammation. Strain-dependent variations in the lipid A moiety dictate the immunologic activity of LPS based on how it interacts with pattern recognition receptors [80,81]. Briefly, LPS-binding proteins bind to circulating LPS and transport it to cluster of differentiation 14 (CD14), a co-receptor of LPS mainly expressed by macrophages and other cells involved in the innate immune response. CD14 facilitates the transfer of LPS to the TLR-4–myeloid differentiation protein (MD)-2 complex. MD-2 is the main binding site of LPS; it contains a hydrophobic pocket that interacts with lipid A acyl chains [81]. Once LPS is inserted into the TLR-4–MD-2 complex, differences in acyl chain number and structure govern whether it will have an antagonistic or agonistic effect on TLR-4 [80]. Bacterial strains associated with metabolic endotoxemia produce agonistic LPS, which induce TLR-4 dimerization and subsequent activation. TLR-4 signaling cascades involve myeloid differentiation primary response 88-dependent and -independent pathways [78,82]. These pathways culminate in the expression of pro-inflammatory mediators (e.g., interleukin-6 and -18, tumor necrosis factor [TNF]) through the activation of NF-κB and interferon regulatory factor 3 (IRF-3). This inflammatory response is accompanied by an increase in ROS and oxidative stress [83], creating a forward-feeding mechanism of cellular damage. In this way, persistent metabolic endotoxemia promotes a state of chronic low-grade inflammation and oxidative stress, both of which are features of many pathophysiological conditions such as insulin resistance, type 2 diabetes, and obesity [84,85,86].

## 5. Oxidative Stress, Gut Microbiota, and p66Shc

The resilience of an organism is tied to its ability to adapt and respond to different stressors both endogenous and exogenous. Rheostasis is a feature of adaptation as the same biochemical processes or components govern different physiological outcomes through continuous regulation. ROS are a classic example of rheostatic activity, governing different cell fates depending on their type, level, and localization [87]. As mentioned in the previous section, transient ROS production in the gut stimulate cellular pathways leading to cell proliferation [70]. Here we contend that another rheostat, p66Shc, may play a role in this process.

Small formylated peptides shed by commensal bacteria and recognized by formyl peptide receptors induce an increase in the activity of NADPH oxidases, thereby increasing local ROS levels [70]. This inactivates certain redox-sensitive proteins such as dual specific phosphatase-3 (DUSP3), a phosphatase involved in the regulation of MAPK/ERK proliferative pathways. When active, DUSP3 dephosphorylates MAPKs and downregulates MAPK/ERK pathways. ROS can oxidize cysteine residues in the DUSP3 catalytic site, rendering it inactive [88]. Thus, commensal bacteria can promote epithelial cell proliferation and gut barrier integrity through enzymatic ROS production.

Mitochondrial dynamics are integral to cellular proliferation, with mitochondrial biogenesis and the replication of mitochondrial DNA (mtDNA) being essential for ensuring adequate energy supply and metabolic function in daughter cells [89,90], and the adaptor protein p66Shc has been implicated in this process. Studies by Trinei and colleagues [91] have demonstrated that p66Shc can upregulate mtDNA replication independently of its established roles in reactive oxygen species (ROS) generation and apoptosis. Remarkably, the absence of p66Shc was correlated with a substantial reduction in mtDNA content by 40–50%, suggesting an unanticipated role for p66Shc in mitochondrial maintenance [91]. This p66Shc-dependent mtDNA replication enhancement, as suggested by Trinei et al. [91], could represent a fundamental step linking mitochondrial function to the broader cellular replication process, given the findings by Blank et al. [92]. They observed that increased mtDNA replication in yeast could initiate nuclear DNA replication and cellular proliferation, suggesting a mechanism where mtDNA replication, potentially regulated by p66Shc, may precede, and facilitate nuclear DNA replication and cellular proliferation in a conserved manner across species. These insights position p66Shc as a potential regulator of mitochondrial distribution and function during cell division, warranting further investigation into its role in mtDNA replication and the consequent phenotypic effects. The implications of p66Shc’s involvement in these processes could offer a novel perspective on the regulation of gut epithelial cell proliferation, an area ripe for exploration considering the critical importance of gut homeostasis in health and disease.

**Figure 3:**
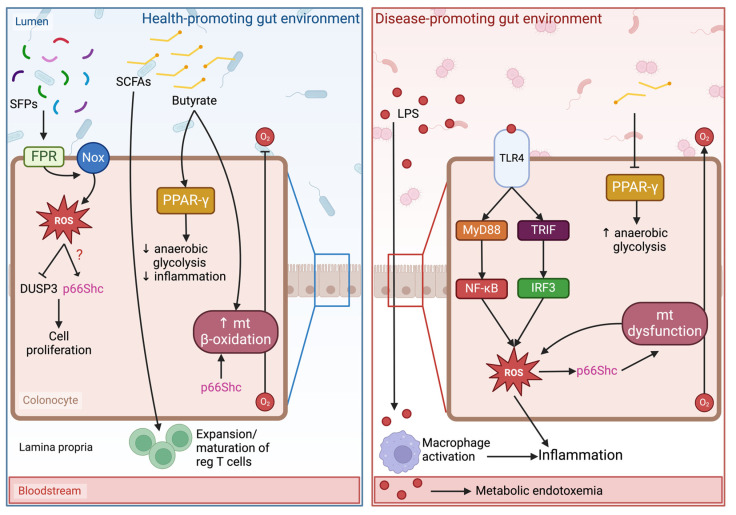
In a health-promoting gut environment (**left panel**), microbial signals promote cell proliferation, immune cell regulation, and oxygen utilization, among other effects not illustrated here. The redox sensor p66Shc may contribute to homeostasis maintenance by supporting cell proliferation and mitochondrial efficiency in response to physiological and transient reactive oxidative species (ROS) production. In a disease-promoting gut environment (**right panel**), microbial signals promote sustained oxidative stress and inflammatory signals. In this scenario, p66Shc amplifies and propagates oxidative damage and mitochondrial dysfunction. SFPs, small formylated peptides; FPR, formylated peptide receptor; Nox, NADPH oxidase; DUSP3, dual specificity protein phosphatase 3; PPAR-γ, peroxisome proliferator-activated receptor gamma; mt, mitochondria; LPS, lipopolysaccharide; TLR4, toll-like receptor 4; MyD88, myeloid differentiation primary response 88; TRIF, TIR-domain-containing adaptor-inducing beta interferon; NF-κB, nuclear factor kappa B; IRF3, interferon regulatory factor 3. Created with BioRender.com.

On the other side of the rheostatic spectrum, p66Shc may also exacerbate gut microbiota-derived signals that are associated with pathophysiological conditions. Locally, SCFAs contribute to colonic homeostasis by stimulating PPAR-γ signaling, inducing regulatory T-cell maturation, and providing fuel for mitochondrial beta-oxidation [74,93]. Specifically, SCFAs bind to G protein-coupled receptor 43 on the surface of colonic T-cells, thus inducing the maturation and expansion of regulatory T-cells [94], which control inflammatory responses in mucosal tissues such as the colon. Consequently, when levels of SCFAs are insufficient, the colonic environment undergoes a shift in metabolism that favors oxidative and inflammatory conditions. PPAR-γ signaling downregulation increases local oxygen concentration and regulatory T-cell depletion induces intestinal inflammation. This combination is a one–two punch to mitochondria as evidenced by a decreased oxygen consumption despite increased local oxygen bioavailability [74]. The oxidative and inflammatory environment described above favor p66Shc’s pro-apoptotic functions; therefore, it is plausible that the electron transport chain disruption observed in these conditions is mediated, at least in part, by p66Shc. The potential rheostatic role of p66Shc and ROS in the local colonic environment is illustrated in Figure 3.

The relationship between the gut microbiota and p66Shc likely goes beyond local effects. As detailed in the previous section, gut microbiota-induced metabolic endotoxemia promotes low-grade chronic inflammation and oxidative stress through activation of NF-κB and IRF-3 pathways mediated by TLR4 activation [78]. This process is associated with the onset of many chronic diseases such as obesity and type 2 diabetes [61,84,85,86,95,96]. As inflammation and oxidative stress are associated with mitochondrial dysfunction, p66Shc has also been implicated in the onset and progression of these diseases (Table 1) [19,97,98,99]. However, as detailed in a recent review by Ciciliot and Fadini [19], the evidence for this association is conflicting.

This seemingly contradictory evidence may be parsed out by considering a missing confounder in these studies: the gut microbiota. To date, only one study has explored the differences between the gut microbiota composition of animals with and without p66Shc ablation [5]; the authors reported that p66Shc knockout altered gut microbiota composition and metabolic output in mice, and that this alteration modulated their phenotypic response to a high-fat diet. However, this study did not utilize a humanized gnotobiotic mouse model, which limits the interpretation of the results in the human context [100,101]. Thus, the complexity of the interaction between the metabolic output of gut microbes and p66Shc remains underexplored.

Herein, we hypothesize that adipose tissue is a major site for the interaction between microbial signals, namely LPS, and p66Shc. Firstly, LPS receptor TLR4 and p66Shc are both highly expressed in adipocytes [102]. Further, LPS-induced TLR4 activation in macrophages leads to adipose tissue infiltration, ultimately leading to secretion of pro-inflammatory cytokines and other inflammatory signals to surrounding tissues [103]. Secondly, available evidence supports a complementary role of LPS and p66Shc in insulin-dependent signaling pathways regulating adipose tissue metabolism. As discussed previously, LPS-induced TLR4 activation stimulates NF-kB signaling, which leads to an increase in the expression of pro-inflammatory cytokines. TNF-alpha is one of these cytokines, and it plays a major role in the onset of insulin resistance [104]. Briefly, TNF-alpha secreted by stressed adipocytes induces phosphorylation of insulin receptor substrate 1 (IRS-1), a critical regulator of insulin signaling, in muscle cells. This phosphorylation inactivates IRS-1 and impairs insulin-dependent downstream cascades [105,106]. Consequently, anabolic nutrient-sensing pathways such as those mediated by insulin and mTORC1 are inhibited.

In addition, LPS-induced inflammation can promote p66Shc activation due to the associated increase in oxidative stress. Ranieri and colleagues reported that p66Shc can inactivate IRS-1 in adipose tissue via an interaction with insulin effector kinase 1 [97,107], thereby contributing to the onset of insulin resistance. These observations were made in the context of high fat-induced obesity, and dietary fatty acids can stimulate TLR4 similarly to LPS [108]. However, because high-fat diets induce changes in the gut microbiota that lead to metabolic endotoxemia [109,110], it is likely that dietary fatty acids affect TLR4 signaling via increased circulating LPS. In sum, LPS and p66Shc can work together to initiate insulin resistance and deregulate nutrient-sensing pathways. This deregulation has important implications for cellular adaptive stress responses such as endogenous antioxidant production and autophagy (including mitophagy), which ultimately lead to phenotypic manifestations and the development of chronic diseases.

## 6. Linking Oxidative Stress, Gut Microbiota, and p66Shc to Pathophysiological Outcomes

The previous sections outlined how the gut microbiota and p66Shc can exacerbate oxidative stress and inflammation. In this section, we will explore the pathophysiological outcomes of these processes and how they can contribute to the onset and progression of chronic diseases. Chronic diseases such as type 2 diabetes and cardiovascular diseases have common risk factors, including hyperglycemia, dyslipidemia, and endothelial dysfunction. These risk factors, in turn, have common etiologies, with the most prominent of them being insulin resistance [111].

Insulin is an essential endocrine hormone involved in glucose homeostasis and anabolic metabolism. Secreted by pancreatic beta cells upon nutrient availability signaling (e.g., exogenous glucose from a meal), insulin increases anabolic pathways while decreasing catabolic pathways. Insulin has systemic effects, with direct and indirect action on important organs and tissues, namely the liver, skeletal muscle, and adipose tissue, among others [112]. Insulin resistance is defined as an impaired response of these targets to insulin stimulation, which leads to hyperglycemia due to decreased glucose utilization and hyperinsulinemia due to compensatory insulin production [111]. These processes start a metabolic derangement that serve as a foundation to many chronic diseases.

Insulin resistance is most commonly initiated by modifiable lifestyle-related risk factors that lead to chronic overnutrition and obesity, such as physical inactivity and poor dietary habits [111]. In particular, diets high in saturated fat and simple carbohydrates (commonly referred to as a Western pattern diet) are most associated with an increased risk of developing insulin resistance and associated diseases [113]. Chronic overnutrition combined with a Western pattern diet induce changes in the microbial metabolic output that favor pro-oxidant and -inflammatory processes and disrupt energy metabolism; the most well-described changes are decreases in SCFAs and secondary bile acids and increases in LPS and branched-chain amino acids [114,115]. In Section 4 and Section 5, we describe how SCFA depletion and LPS-mediated endotoxemia lead to an increase in inflammation via NF-kB signaling and subsequent IRS-1 inactivation, thereby dampening insulin-sensitive metabolism. In white adipose tissues, this dampening of metabolic responses to insulin means that lipogenesis is suppressed while lipolysis continues to be stimulated even in conditions of nutrient abundance, supplying non-adipose tissues with excess nonesterified fatty acids (NEFAs) and impairing lipid storage in adipocytes [116]. Circulating NEFAs are captured by the liver and skeletal muscle, where they can be stored in lipid droplets or utilized as an acetyl-CoA precursor for oxidative phosphorylation in the mitochondria. However, as the supply of NEFAs outpaces the demand for ATP, excessive β oxidation leads to ROS overproduction [117,118] and mitochondrial dysfunction [119], thus perpetuating and amplifying stress-mediated IRS inactivation and insulin resistance.

**Figure 4:**
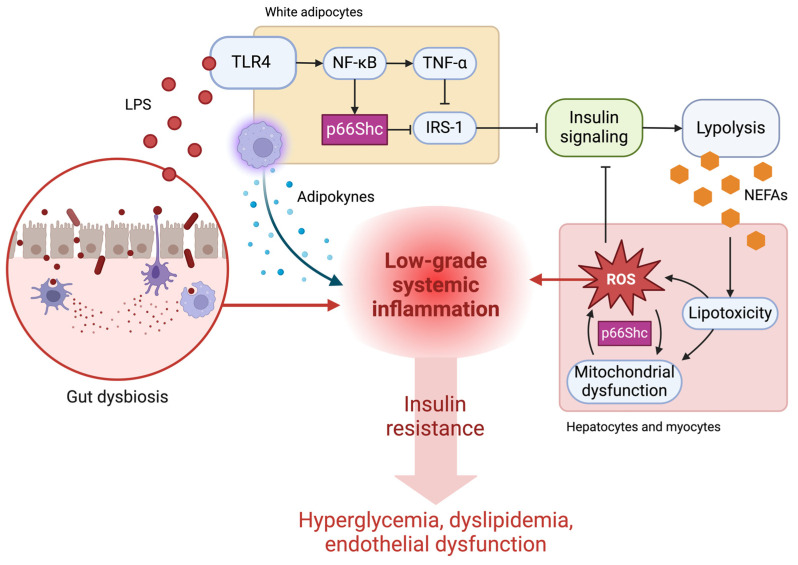
The adaptor protein p66Shc may mediate the effect of microbial signals in distal tissues and play a role in the gut microbiota–mitochondria axis. Activation of pro-inflammatory signals in white adipocytes by lipopolysaccharides (LPS) can activate the pro-oxidant function of p66Shc and further contribute to the dampening of insulin signaling. Insulin-resistant adipocytes continue to break down triacylglycerols and secrete non-esterified fatty acids (NEFAs). Excess NEFAs induce lipotoxicity in the liver and skeletal muscle, damaging mitochondria and contributing to insulin resistance in these organs. Insulin resistance underlies the etiology of many chronic diseases. TLR4, toll-like receptor 4; NF-κB, nuclear factor kappa B; TNF-α, tumor necrosis factor alpha; IRS-1, insulin receptor substrate 1. Created with BioRender.com.

Available evidence indicates that p66Shc participates in the gut–mitochondria axis described above and illustrated in Figure 4. Studies have reported that p66Shc inhibits insulin-dependent anabolic metabolism [107,120,121] via IRS-1 inactivation, indicating that oxidative stress resulting from gut metabolite signaling can activate p66Shc and worsen insulin sensitivity. However, whether p66Shc antagonizes insulin through its effect on mitochondria or through its own enzymatic ROS production remains unclear. Further, in normal physiological conditions, Berniakovich and colleagues [98] reported that p66Shc couples insulin signaling to mitochondrial respiration in adipose tissue, increasing lipogenesis and decreasing fatty acid oxidation in adipocytes. As the p66Shc knockout mice in this study were reported to have less energy storage and increased energy expenditure than their counterparts, it is possible that the apparent longevity conferred to these mice by knocking out p66Shc would not occur in natural life scenarios, indicating an evolutionary adaptation. Interestingly, Ciciliot and colleagues [99] reported that p66Shc deletion had no protective effect on insulin resistance in mice fed a high-fat diet. In a complementary study, Ciciliot and collaborators [5] showed that the gut microbiota of p66Shc-knockout mice had a worse metabolite output than their wild-type counterparts even when fed a standard diet. This observation ties back to the argument described herein on the involvement of p66Shc in gut epithelial cell proliferation under normal physiological conditions (Section 5), which is paramount for a health-promoting intestinal environment. Another possibility is that p66Shc deletion by itself cannot protect cells and tissues from the systemic and propagative injuries associated with insulin resistance (see Ref. [112] for an exhaustive review on the mechanisms of insulin resistance).

The events outlined in this section are just a part of the gut–mitochondria axis and illustrate how gut metabolites initiate a cascade of highly integrated pathways that lead to mitochondrial dysfunction and pathophysiological states. In the context of chronic diseases, gut and mitochondrial signals seem to converge in the adipose tissue, meriting further investigation. As a major endocrine organ, the pro-oxidant and -inflammatory signals transmitted by dysfunctional adipose tissue are amplified and contribute to the systemic low-grade inflammation that typifies obesity and insulin resistance. Moreover, the burgeoning field of pharmaco-microbiomics has begun to unravel complex interactions between pharmacological agents, the gut microbiota, and mitochondrial function. Certain medications have been shown to influence gut microbiota composition, which in turn can affect mitochondrial dynamics via metabolic signaling pathways, potentially modulating the activity of p66Shc. This intricate network underscores the potential of targeting the gut–microbiota–mitochondrial axis, with p66Shc as a critical node, to develop innovative therapeutic strategies for diseases underscored by mitochondrial dysfunction. Emphasizing the significance of these relationships offers promising avenues for clinical research, aiming to harness the regulatory potential of p66Shc within the mitochondria to ameliorate or prevent the progression of related pathologies.

## 7. Conclusions and Future Directions

This review elucidates p66Shc as a potential mediator for crosstalk between the gut microbiota and mitochondrial function, a relationship integral to cellular response to oxidative stress and pathogenesis of metabolic diseases. The evidence presented highlights the complexity of this interaction, where p66Shc modulates key signaling pathways implicated in both apoptotic initiation and inhibition, as well as regulation of antioxidative defenses. Insights garnered from this synthesis suggest a significant therapeutic potential in targeting the gut microbiota–p66Shc–mitochondrial axis, particularly through non-pharmacological interventions such as dietary modulation. These strategies may offer a promising avenue for mitigating the deleterious effects of mitochondrial dysfunction in metabolic diseases. Further studies into molecular mechanisms underpinning microbial metabolite-mediated modulation of p66Shc activity are needed. Such investigations are vital for developing targeted interventions. In parallel, robust clinical studies are needed to establish causal relationships and support the translational potential of preclinical findings. Furthermore, embracing gut microbiota diversity across populations and individual genetic variability will be critical for advancing personalized medical approaches. As research progresses, the integration of advanced in vivo models and biotechnological innovations will be crucial for translating these complex biological interactions into tangible therapeutic modalities.

## Figures and Tables

**Table 1:** Selected studies published in the last 10 years investigating the role of p66Shc on different pathophysiological conditions.

**First Author**	**Year**	**Study Population or Model**	**Pathophysiological Condition**
W. E. Hughes [21]	2021	Animal (rats)	Hypertension
K. Shahzad [22]	2018	Animal (mice)	Hyperglycemia-induced atherosclerosis
S. Costantino [23]	2018	Animal (mice) Cell culture (human cardiomyocytes)	Diabetes-related cardiomyopathy
H. Vashistha [24]	2018	Animal (mice) Cell culture (Sca-1+ mesenchymal stem cells)	Diabetes-related renal dysfunction
F. Paneni [25]	2016	Cell culture (early outgrowth cells)	Age-related impaired vascular repair
R. Vono [26]	2016	Humans (patients with diabetes undergoing major limb amputation)	Diabetes-related critical limb ischemia
A. Akhmedov [27]	2015	Animal (mice)	Cardiac ischemia and reperfusion
R. D. Spescha [28]	2015	Animal (mice) Cell culture (primary HBMVECs) Human (acute ischemic stroke patients)	Ischemia/reperfusion brain injury; stroke
A. Natalicchio [29]	2015	Animal (mice) Cell culture (rat INS-1E cells; murine, human, and mouse islets)	Hyperglycemia
Y. Shi [30]	2014	Animal (mice)	Age-related cerebrovascular impairment
F. Paneni [31]	2014	Animal (mice)	Obesity-induced endothelial insulin resistance
R. D. Spescha [32]	2014	Cell culture (primary human AECs and rat AECs)	Hypertension
A. Vikram [33]	2014	Animal (mice) Cell culture (various)	Endothelial dysfunction
Z. Chen [34]	2014	Animal (mice) Cell culture (Caco-2 cells)	Ischemia/reperfusion intestinal injury
V. Bellisario [35]	2014	Animal (mice)	Detrimental developmental programming
R. D. Spescha [36]	2013	Animal (mice)	Ischemia/reperfusion brain injury; stroke
L. Laviola [37]	2013	Cell culture (HUVECs)	Endothelial dysfunction
F. Bock [38]	2013	Animal (mice)	Diabetes-related nephropathy

AECs, aorta endothelial cells; HBMVECs, human brain microvascular endothelial cells; HUVECs, human umbilical vein endothelial cells.

**Table 2:** Selected studies published in the last 10 years investigating associations between the gut microbiota and different pathophysiological conditions.

**First Author**	**Year**	**Study Population**	**Pathophysiological Condition**
J. A. Larke [58]	2023	Healthy adults	Gastrointestinal inflammation
Y. Ikubo [59]	2022	Patients with CTEPH	Pulmonary hypertension
R. L. Walker [60]	2021	Framingham Heart Study cohort	Cardiometabolic diseases
T. V. Rohm [61]	2021	Obese and non-obese adults	Obesity
X. Wang [62]	2020	Patients with ESRD	Renal disease
Y. Wan [63]	2020	Adults in different BMI categories	Cardiometabolic diseases
F. Piñero [64]	2019	Patients with cirrhosis	Liver cancer
J. Shapiro [65]	2019	Patients with psoriasis	Autoimmune diseases
M. Trøseid [66]	2015	Patients with chronic heart failure	Cardiovascular diseases
M. Rossi [67]	2014	Patients with CKD	Renal disease

CTEPH, chronic thromboembolic pulmonary hypertension; BMI, body mass index; ESRD, end-stage renal disease; CKD, chronic kidney disease.

## Data Availability

No new data were created or analyzed in this study. Data sharing is not applicable to this article.

## Abbreviations

MAPK—Mitogen-Activated Protein Kinase; PTB—Phosphotyrosine-Binding; CH—Collagen Homology; SH2—Src Homology 2; Grb2—Growth Factor Receptor-Bound Protein 2; Sos1—Son of Sevenless Homolog 1; FoxO—Forkhead Box O; Akt—Protein Kinase B; PP2A—Protein Phosphatase 2A; SCFA—Short-Chain Fatty Acid; LPS—Lipopolysaccharides; TLR—Toll-Like Receptor; PPAR-γ—Peroxisome Proliferator-Activated Receptor Gamma; CD14—Cluster of Differentiation 14; MD-2—Myeloid Differentiation Factor 2; IRF-3—Interferon Regulatory Factor 3; TNF—Tumor Necrosis Factor; DUSP3—Dual Specificity Phosphatase 3; mtDNA—Mitochondrial DNA; IRS-1—Insulin Receptor Substrate 1; NEFA—Non-Esterified Fatty Acids.
